# Optimization of a Novel Peptide Ligand Targeting Human Carbonic Anhydrase IX

**DOI:** 10.1371/journal.pone.0038279

**Published:** 2012-05-31

**Authors:** Shoaib Rana, Felix Nissen, Annabell Marr, Annette Markert, Annette Altmann, Walter Mier, Juergen Debus, Uwe Haberkorn, Vasileios Askoxylakis

**Affiliations:** 1 Department of Radiation Oncology, University of Heidelberg, Heidelberg, Germany; 2 Clinical Cooperation Unit Nuclear Medicine, German Cancer Research Center, Heidelberg, Germany; 3 Department of Nuclear Medicine, University of Heidelberg, Heidelberg, Germany; University of South Florida College of Medicine, United States of America

## Abstract

**Background:**

Carbonic anhydrase IX (CA IX) is a hypoxia-regulated transmembrane protein over-expressed in various types of human cancer. Recently, a new peptide with affinity for human carbonic anhydrase IX (CaIX-P1) was identified using the phage display technology. Aim of the present study is to characterize the binding site in the sequence of CaIX-P1, in order to optimize the binding and metabolic properties and use it for targeting purposes.

**Methodology/Principal Findings:**

Various fragments of CaIX-P1 were synthesized on solid support using Fmoc chemistry. Alanine scanning was performed for identification of the amino acids crucial for target binding. Derivatives with increased binding affinity were radiolabeled and *in vitro* studies were carried out on the CA IX positive human renal cell carcinoma cell line SKRC 52 and the CA IX negative human pancreatic carcinoma cell line BxPC3. Metabolic stability was investigated in cell culture medium and human serum. Organ distribution and planar scintigraphy studies were performed in Balb/c *nu*/*nu* mice carrying subcutaneously transplanted SKRC 52 tumors.

The results of our studies clearly identified amino acids that are important for target binding. Among various fragments and derivatives the ligand CaIX-P1-4-10 (NHVPLSPy) was found to possess increased binding potential in SKRC 52 cells, whereas no binding capacity for BxPC3 cells was observed. Binding of radiolabeled CaIX-P1-4-10 on CA IX positive cells could be inhibited by both the unlabeled and the native CaIX-P1 peptide but not by control peptides. Stability experiments indicated the degradation site in the sequence of CaIX-P1-4-10. Biodistribution studies showed a higher *in vivo* accumulation in the tumor than in most healthy tissues.

**Conclusions:**

Our data reveal modifications in the sequence of the CA IX affine ligand CaIX-P1 that might be favorable for improvement of target affinity and metabolic stability, which are necessary prior to the use of the ligand in clinical approaches.

## Introduction

Decreased oxygen concentration in solid tumors is known to cause molecular and phenotypic changes that lead to aggressive and rapid tumor growth, as well as to poor responsiveness to chemo- and radiotherapy [1], [2]. At the molecular level hypoxia induces the accumulation of HIF-1α that further activates a series of target genes, including carbonic anhydrase IX (CA IX) [3], [4]. CA IX is a membrane-associated member of the CA zinc metalloenzyme family, known to be involved in tumorigenic processes [5]. The protein is over-expressed in various human tumors and its expression is associated with a poor prognosis [6]–[8]. The fact that CA IX is over-expressed in tumors under hypoxic conditions makes CA IX an attractive candidate for the development of tumor hypoxia targeting approaches.

Prominent examples of molecules with affinity for human carbonic anhydrase IX are sulfonamide motifs and monoclonal antibodies. Sulfonamides can bind CA IX through an interaction with the active center of the enzyme, however their binding is characterized by low specificity due to the high homology of the active centers of different members of the carbonic anhydrase family [9]. Furthermore, although monoclonal antibodies targeting CA IX have been developed and successfully tested in preclinical and clinical trials [10]–[12], antibodies often possess unfavourable pharmacokinetic properties due to their large molecular size.

Peptides present an attractive alternative to antibodies. Due to their small size peptides have improved pharmacokinetic properties, characterized by higher effectiveness of tumor penetration and rapid blood clearance. Furthermore, peptides do not possess the immunogenic potential of antibodies, while they are easier and cheaper to synthesize [13].

Recently we identified a new peptide with specificity for the extracellular domain of CA IX using the phage display technology. The peptide CaIX-P1 (YNTNHVPLSPKY) was identified on the immobilized extracellular domain of CA IX using a commercial available Ph.D.12 library [14]. CaIX-P1 was chemically synthesized and tested *in vitro* for binding affinity, specificity, kinetics and internalization. In addition, the *in vivo* behavior of CaIX-P1 was investigated in biodistribution studies and *in vivo* blocking experiments. The results of these studies indicated a specific binding to human CA IX. Our studies showed a higher accumulation on the immobilized extracellular domain of CA IX and on cells strongly over-expressing the target protein. Peptide binding to the negative control protein EGFR and to negative control cell lines was reduced to the background level. Further evidence for specific target binding was found in competition experiments revealing that the uptake of the radiolabeled ligand could be competitively abolished by the unlabeled peptide but not by unspecific competitors. Furthermore, organ distribution studies in nude mice indicated a higher accumulation in the tumor than in most healthy organs [14], whereas *in vivo* blocking using the unlabeled peptide as competitor led to a significant decrease of radioligand accumulation. These results indicate that CaIX-P1 is an attractive candidate for the development of ligands specifically targeting human CA IX and allow the hypothesis that the peptide might find application for the improvement of hypoxia targeting strategies.

However, the transfer of new peptides to clinical applications is often difficult. One major drawback is the metabolic instability, which results in serum degradation, decreased tumor to organ ratios and enhanced background activity. Therefore, further investigation of the ligand is necessary, prior to its use for clinical application. In this direction identification of the binding region in the peptide sequence is of high importance since it could lead to smaller derivatives with increased tumor penetration and target affinity, allowing at the same time targeted modifications for optimization of the metabolic stability. Aim of the present study is the optimization of the properties of CaIX-P1. To identify the binding amino acids alanine scanning was performed and various fragments of the peptide were synthesized and tested for affinity and specificity on human cancer cells over-expressing CA IX. A derivative that was identified to possess improved binding characteristics was further evaluated *in vitro* and *in vivo*. The results of our study indicate molecular modifications that are necessary for optimization of CA IX targeting strategies and reveal challenges associated with optimizing phage-display derived peptides.

## Results

### CA IX expression in SKRC 52 and BxPC3 cells

The known CA IX positive renal cell carcinoma cell line SKRC 52 and the CA IX negative pancreatic carcinoma cell line BxPC3 were used as positive and negative control target. Prior to the binding experiments, the expression of CA IX in the two cell lines was evaluated using real time PCR and western blot analysis. The analysis demonstrated an expression of CA IX in SKRC 52 cells but no expression in BxPC3 cells (Figure S1).

### Radiolabeling of peptides

The shift in retention times between the labeled and the unlabeled peptides facilitated separation of the tracer with high specific activity. As a result specific activities of approximately 50 GBq/µmol for the ^125^I- and ^131^I- labeled ligands could be obtained.

### Identification of the binding site in the sequence of CaIX-P1

**Figure 1:**
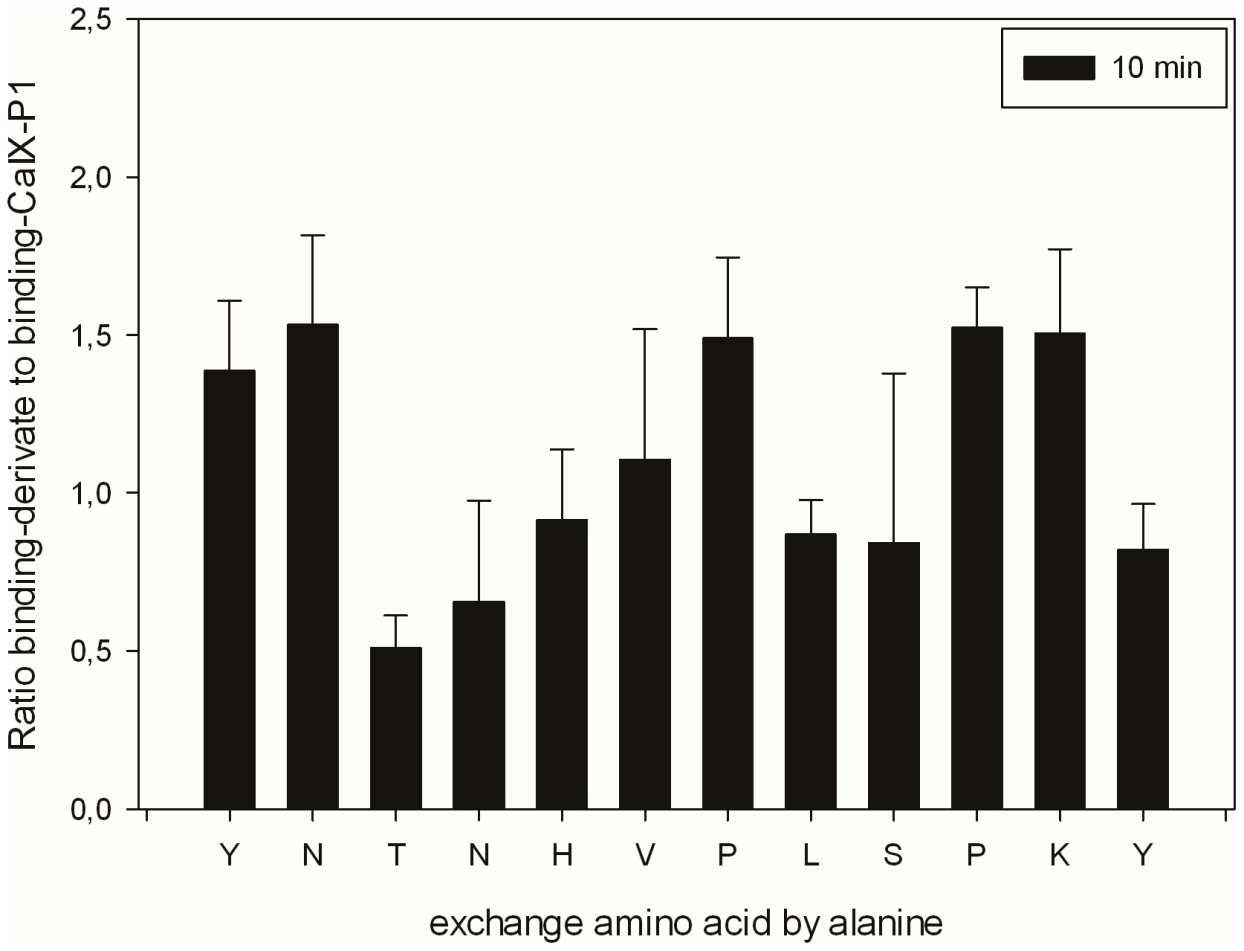
Alanine scanning of ^125^I-labeled CaIX-P1 peptide on CA IX positive human renal cell carcinoma SKRC 52 cells. Ratio binding-derivative to binding-CaIX-P1. Mean values and standard deviation.

To identify the binding site in the sequence of CaIX-P1 and determine which amino acids are responsible for target affinity, alanine scanning was performed. For alanine scanning, derivatives of CaIX-P1 were synthesized with exchange of each amino acid by alanine. All peptide-derivatives were labeled with ^125^I and tested for binding in comparison to the radiolabeled native CaIX-P1 on CA IX positive renal cell carcinoma SKRC 52 cells (Figure 1).

**Figure 2:**
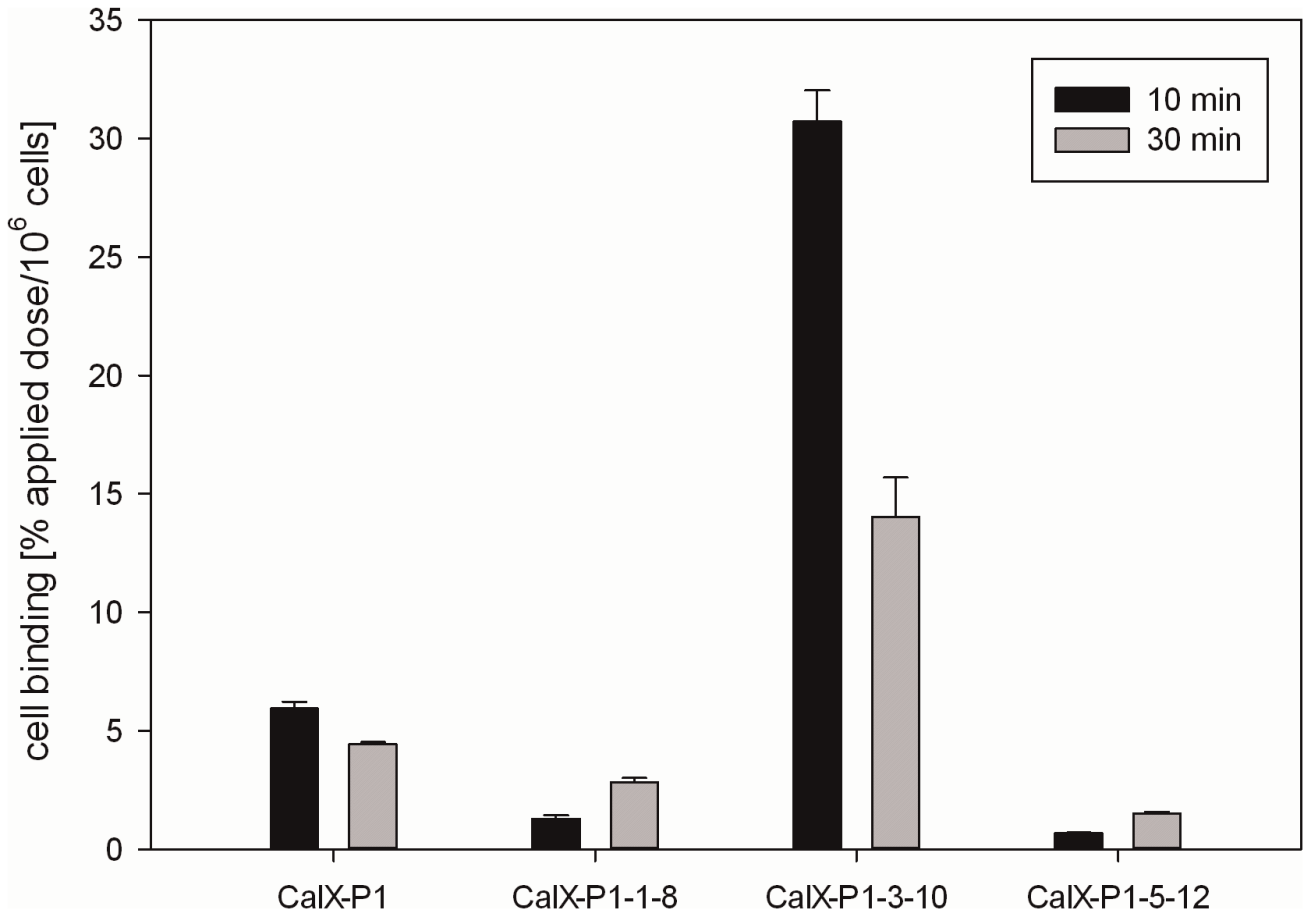
Binding of the CaIX-P1 peptide fragments CaIX-P1-1-8, CaIX-P1-3-10 and CaIX-P1-5-12 on CA IX positive human renal cell carcinoma SKRC 52 cells. Mean values and standard deviation.

The results of alanine scanning showed a significant binding decrease when ^3^T, ^4^N, ^8^L and ^12^Y (p<0.05) were exchanged by alanine. Furthermore, a trend to a binding decrease was noticed for the amino acids ^5^H, ^8^L and ^9^S. These results indicate that the amino acids ^3^T, ^4^N, ^5^H, ^8^L, ^9^S and ^12^Y might be important for CaIX-P1 binding on SKRC 52 cells. To prove this hypothesis, 8-amino acid fragments of CaIX-P1 were synthesized, labeled with ^125^I and investigated for binding on target cells. Octapeptides were chosen because the peptide CaIX-P1-3-10, representing the middle part of CaIX-P1 is the smallest derivative containing all amino acids that were considered by alanine scanning to be important for ligand binding. Comparison after binding saturation of the ^125^I-labeled fragments CaIX-P1-1-8 (YNTNHVPL), CaIX-P1-3-10 (TNHVPLSPy) and CaIX-P1-5-12 (HVPLSPKY) with the native CaIX-P1 (YNTNHVPLSPKY) peptide revealed that the fragment CaIX-P1-3-10 had an up to five-fold higher binding capacity on SKRC 52 cells compared to the leader peptide (p<0.05). The fragments CaIX-P1-1-8 and CaIX-P1-5-12 showed a significantly reduced binding activity (Figure 2). For the native CaIX-P1 and CaIX-P1-3-10 peptide binding was higher after 10 minutes as compared to 30 minutes incubation (Figure 2).

To further investigate the binding site in the sequence of CaIX-P1, several peptide fragments were synthesized and tested on SKRC 52 cells. The ratios binding derivative to binding CaIX-P1 are presented in Table 1.

**Table 1:** Various fragments of CaIX-P1 were tested on CA IX positive human renal cell carcinoma SKRC 52 cells.

Peptide	Sequence	Ratio binding-derivative to binding-CaIX-P1 (10 min)
CaIX-P1	YNTNHVPLSPKY	1
CaIX-P1-3-6	TNHVy	0.29
CaIX-P1-3-7	TNHVPy	0.60
CaIX-P1-3-8	TNHVPLy	0.76
CaIX-P1-3-9	TNHVPLSy	1.57
CaIX-P1-3-10	TNHVPLSPy	5.40
CaIX-P1-4-7	NHVPy	0.79
CaIX-P1-4-8	NHVPLy	0.82
CaIX-P1-4-9	NHVPLSy	2.62
CaIX-P1-4-10	NHVPLSPy	5.84
CaIX-P1-5-9	HVPLSy	0.06
CaIX-P1-5-12	HVPLSPKy	0.10
CaIX-P1-6-10	VPLSPy	0.25
CaIX-P1-8-10	LSPy	0.04

Ratio binding-fragment to binding-CaIX-P1.

These data indicate that the sequence CaIX-P1-4-10 represents the shortest derivative with the highest binding capacity on the CA IX over-expressing cells. CaIX-P1-4-10 had an almost six-fold binding on SKRC 52 cells compared to the native CaIX-P1 peptide (p<0.05), reaffirming the previous hypothesis that the binding site in the sequence of CaIX-P1 might be between ^3^Thr and ^10^Pro.

### *In vitro* and *in vivo* evaluation of CaIX-P1-4-10

**Figure 3:**
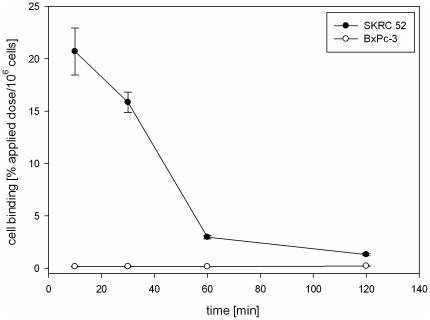
*In vitro* kinetics of ^125^I-CaIX-P1-4-10 on SKRC 52 and BxPC3 cells. Incubation was performed for time periods varying from 10 min to 120 min. Mean values and standard deviation.

Since the peptide CaIX-P1-4-10 showed a significantly higher binding than CaIX-P1, *in vitro* kinetics, competition and internalization studies, as well as *in vivo* distribution experiments were performed. Binding kinetics of ^125^I-CaIX-P1-4-10 in SKRC 52 cells, with incubation periods varying from 10 min to 2 h, revealed a maximal binding of about 21% applied dose/10^6^ cells after 10 min incubation. Thereafter a time-dependent decrease was noticed with the bound activity reaching a value of about 1.3% after 2 h incubation. No binding was measured on CA IX negative BxPC3 cells (Figure 3).

**Figure 4:**
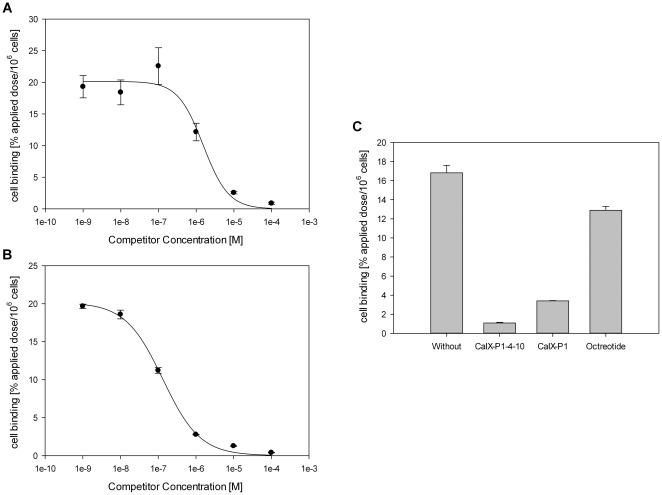
Displacement of bound ^125^I-CaIX-P1-4-10 by the unlabeled CaIX-P1 (A) and the unlabeled CaIX-P1-4-10 (B) peptide at various concentrations on SKRC 52 cells. (C) Specific binding of ^125^I-CaIX-P1-4-10 on SKRC 52 cells. Non specific binding was determined in the presence of 10^−5^ M unlabeled CaIX-P1-4-10 and CaIX-P1. Octreotide was used at the same concentration (10^−5^ M) as a negative control competitor. Mean values and standard deviation.

To further verify the binding affinity and specificity, competition studies were carried out. Co-incubation of ^125^I-CaIX-P1-4-10 with unlabeled CaIX-P1-4-10 or unlabeled CaIX-P1 on SKRC 52 cells resulted in a concentration dependent inhibition of the radioligand binding with an inhibition of over 95% at 10^−4^ mol/L competitor concentration (p<0.05) (Figure 4A, 4B). The IC_50_ values were calculated to be 1.3×10^−7^ M when CaIX-P1-4-10 was used as competitor and 1.5×10^−6^ M when CaIX-P1 was used as competitor. At a competitor concentration of 10^−5^ mol/L CaIX-P1-4-10 showed a binding inhibition of about 95% and CaIX-P1 of about 80% (p<0.05). The negative control competitor octreotide caused only a slight inhibition of radioligand binding (Figure 4C).

### Internalization experiments of CaIX-P1-4-10

**Figure 5:**
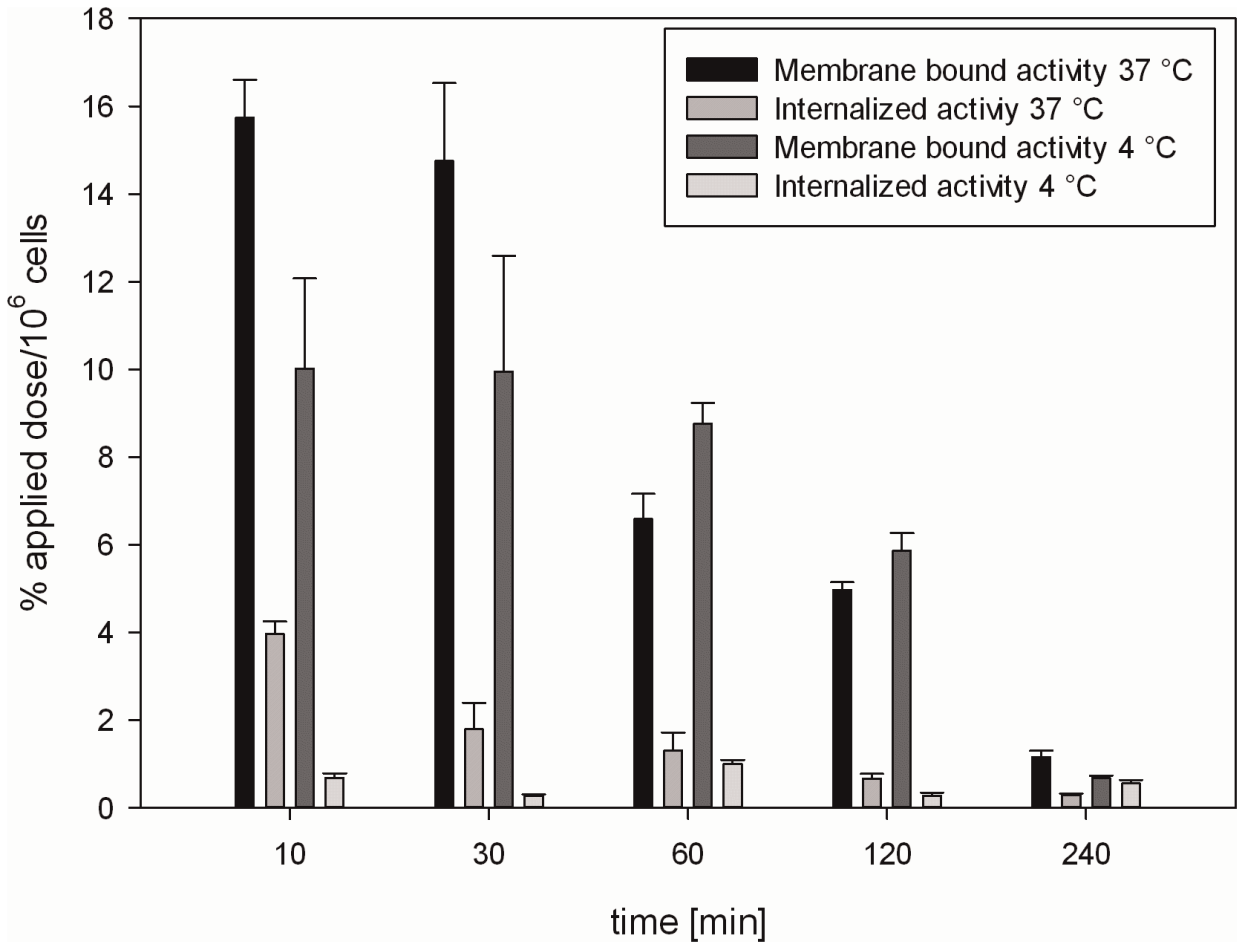
Binding and internalization of ^125^I-CaIX-P1-4-10 in SKRC 52 cells. Cells were incubated with the radioligand for 10 min, 30 min, 60 min, 120 min and 240 min at 37°C or at 4°C. Mean values and standard deviation.

To distinguish between surface bound and internalized peptide, *in vitro* internalization was investigated in SKRC 52 cells. After 10 min incubation with ^125^I-CaIX-P1-4-10 at 37°C, the internalized radioactivity was measured to reach the level of about 20% of the total bound activity, while after 60 min incubation 17% of the total uptake was found to be internalized into the SKRC 52 cells. With time progression both membrane bound and internalized radioactivity decreased. Internalization experiments were also performed at 4°C demonstrating a significant reduction of internalized activity, leading to reduction of total binding (p<0.05) (Figure 5).

### Organ distribution studies of CaIX-P1-4-10

**Figure 6:**
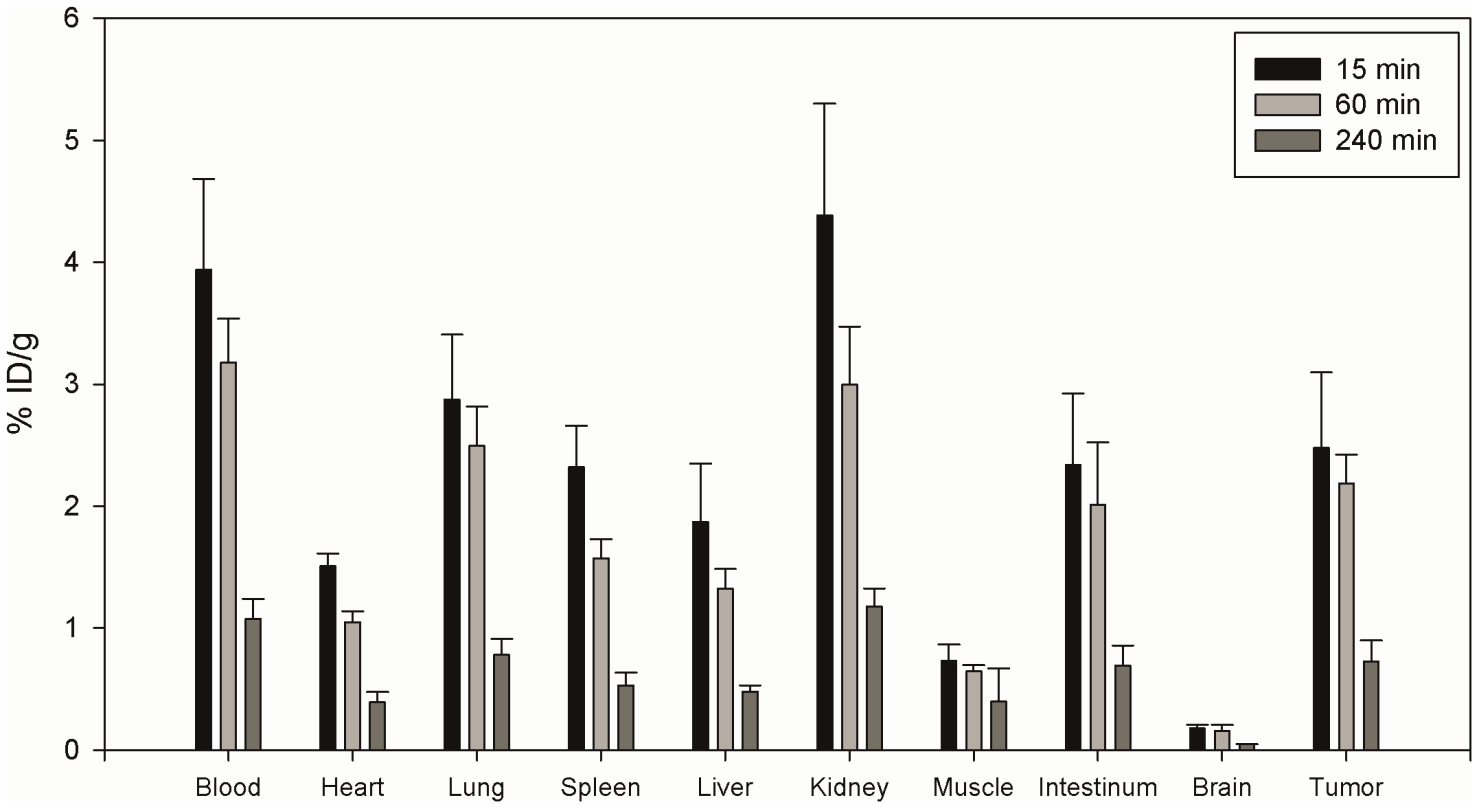
Organ distribution of ^131^I-labeled CaIX-P1-4-10 in female Balb/c nu/nu mice carrying SKRC 52 tumors. Incubation was performed for 15 min, 60 min and 240 min (3 animals per experiment).

Biodistribution studies of ^131^I-labeled CaIX-P1-4-10 were performed in nude mice, carrying subcutaneous SKRC 52 tumors. The results of these studies showed a higher uptake in the tumor than in most healthy organs, such as heart, spleen, liver, muscle and brain after 15 min and 60 min circulation in the animals. Increased activities were measured for blood and kidney. Between 15 min and 60 min a binding decrease was noticed in both tumor and healthy tissues. The decrease in the tumor was lower compared to the other organs, resulting in an increase of the tumor to organs ratios (Table 2). Thereafter, a strong decrease in all organs was noticed (Figure 6).

**Table 2:** Tumor-to-organ ratios calculated from the organ distribution of ^131^I-CaIX-P1-4-10 in female Balb/c mice carrying subcutaneously transplanted SKRC 52 tumors (n = 3 animals per time point).

Tumor to organ ratio	15 min	60 min	240 min
Blood	0.63	0.69	0.67
Heart	1.63	2.09	1.84
Lung	0.86	0.88	0.92
Spleen	1.06	1.39	1.35
Liver	1.32	1.65	1.49
Kidney	0.56	0.73	0.61
Muscle	3.36	3.39	2.53
Brain	13.87	14.91	15.10

### Planar scintigraphy

**Figure 7:**
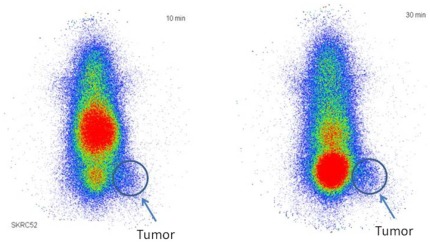
Planar scintigraphy of ^125^I-CaIX-P1-4-10 in SKRC 52 tumor bearing mice. Tumors were transplanted at the right hind leg of the animals (n = 2). Images were obtained at 10 min and 30 min.

*In vivo* planar scintigraphy studies were performed in nude mice, carrying subcutaneously SKRC 52 tumors in the upper part of the hind leg. Planar scintigraphy showed a tumor visualization at 10 min p.i. Furthermore, the planar images demonstrated a rapid renal elimination but also a high background noise (Figure 7).

### Stabilization of ^125^I-labeled CaIX-P1-4-10 on SKRC 52 cells

**Figure 8:**
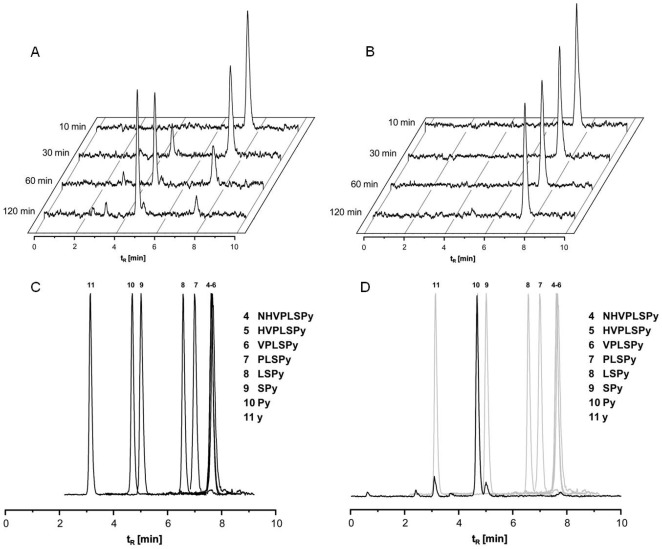
*In vitro* stability studies of ^125^I-CaIX-P1-4-10 on (A) SKRC 52 cells and (B) BxPC3 cells. (C) HPLC chromatograms of all C-terminal fragments of CaIX-P1-4-10. (D) HPLC Chromatogram of C-terminal fragments of CaIX-P1-4-10 and HPLC Chromatogram of supernatant in cell media of SKRC 52 cells are superimposed.

The decreasing binding kinetics of the native CaIX-P1 peptide was explained in the initial study by an intracellular degradation of the peptide [14]. To investigate this hypothesis for CaIX-P1-4-10 the medium stability of this peptide was investigated after incubation on SKRC 52 and BxPC3 cells. Medium stability studies demonstrated a time dependent degradation of CaIX-P1-4-10 on SKRC 52 cells. In particular, only 30% of the activity was measured after 60 min incubation, while at 120 min incubation radiolabeled CaIX-P1-4-10 was completely degraded (Figure 8A). No radioligand degradation was shown in CA IX negative BxPC3 cells (Figure 8B).

To evaluate the degradation site in the sequence of CaIX-P1-4-10, HPLC chromatograms of all C-terminal fragments of the peptide were analyzed (Figure 8C).

The HPLC analysis of the C-terminal fragments of CaIX-P1-4-10 was compared to the chromatograms of the cell medium supernatants. This comparison revealed that the peptide was degraded between ^6^S and ^7^P and the last fragment was Py (Figure 8D).

### Stability in human serum

Metabolic stability of CaIX-P1-4-10 was also investigated in human serum. The results of serum stability experiments revealed slow time-dependent peptide degradation. Serum half life of CaIX-P1-4-10 was calculated to be 1.5 h.

## Discussion

Carbonic anhydrase IX (CA IX) is a transmembrane protein, linked to cancer development [15]–[18] and considered to be an intrinsic marker of tumor hypoxia. The protein distribution in human tissues exhibits a unique pattern that enables designation of CA IX as a tumor-associated protein [19], making it an attractive structure for specific targeting approaches.

Recently, we identified a new peptide (CaIX-P1) with affinity for the extracellular domain of human CA IX using phage display [14]. The peptide evaluation indicated that CaIX-P1 might be an attractive ligand for targeting CA IX in tumors. Aim of the present study is to further characterize the CaIX-P1 peptide, identify the amino acids that are essential for target binding and optimize its affinity and metabolic characteristics.

To achieve these goals a systematic approach was applied. First, alanine scanning was performed in order to identify single amino acids that might be important for target binding. Thereafter, various derivatives and fragments were synthesized and tested for binding. The derivative that showed the highest binding capacity was evaluated for binding kinetics, competition, internalization and biodistribution, as well as for metabolic stability using the well established, CA IX over-expressing cell line SKRC 52 [20].

Our results indicate that the amino acids ^3^T, ^4^N, ^5^H, ^8^L, ^9^S and ^12^Y are important for the target affinity of CaIX-P1. This hypothesis is supported by the evaluation of three octapeptides derived from the sequence of CaIX-P1, demonstrating that the fragment CaIX-P1-3-10, which includes all these amino acids had a strongly increased binding affinity. In contrary, fragments that lacked more than one of these amino acids showed a significant binding decrease. These results are in concert with further studies showing that peptide fragments lacking more than 2 amino acids, indicated to be essential, had a significantly decreased binding capacity.

Since ^12^Y is indicated to be important for ligand binding, an issue that needs to be addressed is the influence of radioiodination on the properties of the peptide. The addition of one radioiodine atom to large molecules, such as antibodies does not usually affect their activity, mainly because the iodine substituent is too small to induce significant conformational changes [21]. However, the incorporation of an iodine atom in small peptides may have negative effects on their binding properties, especially for oligopeptides. Consequently, the pharmacokinetic properties of each individual ligand should be further investigated to characterize the functional integrity of the ligands after iodination.

Characterization of various peptide fragments revealed a molecule consisting of 7 amino acids and a C-terminal D-tyrosine residue with the highest binding capacity (CaIX-P1-4-10). To determine the characteristics of the best binder a detailed evaluation was performed. *In vitro* experiments demonstrated an enhanced accumulation of CaIX-P1-4-10 on CA IX positive cells but no binding on CA IX negative cells. However, kinetic studies showed a time dependent binding decrease. Similar kinetics have been described for both the native CaIX-P1 peptide [14], but also for monoclonal antibodies targeting human carbonic anhydrase IX [22]. The time dependent binding reduction was explained by processes of degradation, leading to radioactive products that are excreted by the cells. To prove this hypothesis internalization experiments and HPLC analysis of cell culture medium after radioligand incubation on CA IX positive and CA IX negative cells were performed. The internalization experiments revealed a slight cellular uptake, whereas the HPLC chromatograms demonstrated a peptide degradation. No degradation was noticed when the peptide was incubated with CA IX negative cells. These results together strengthen the hypothesis of a peptide uptake in CA IX positive cells, followed by cellular degradation and externalization of radiolabeled products, which results in a time dependent activity decrease.

Evidence for the specificity of CaIX-P1-4-10 was shown in competition experiments. These studies revealed a concentration dependent inhibition of radioligand binding, when both the unlabeled CaIX-P1-4-10 peptide, but also the native CaIX-P1 ligand was used as competitor. The binding specificity was further indicated by experiments revealing no reduction of radioligand binding when the negative control competitor octreotide was used.

*In vivo* distribution of CaIX-P1-4-10 showed a higher accumulation in tumor than in most healthy tissues, but also demonstrated increased background levels, result which correlated with planar scintigraphy images. Planar scintigraphy indicated a visualization of the tumor but also demonstrated a rapid renal elimination. High background noise is a major drawback for the use of ligands for imaging applications. The metabolic properties of the peptide are essential in this respect. In particular, most linear peptides reveal short serum half lives, as a result of rapid proteolytic degradation. Metabolic instability leads to circulation of products that either lack target affinity or inhibit further ligand binding. Investigation of the metabolic properties of CaIX-P1-4-10 revealed an increased stability in human serum (serum half life 1.5 h) compared to the native CaIX-P1 peptide (serum half life 25 min). However, our results indicated a rapid cellular degradation followed by radioactivity externalization. The combination of rapid cellular degradation and serum instability might be responsible for the high blood background. Therefore, further improvement of the metabolic properties of CaIX-P1-4-10 is still necessary. Possible chemical methods that can be applied in this respect include peptide cyclization [23], targeted exchange of amino acids with D-amino acids, which are not recognized by serum proteases [24] or grafting the binding motif into a stable scaffold structure [25].

In conclusion, our study reveals modifications in the sequence of the human carbonic anhydrase IX binding peptide CaIX-P1 that might increase its target affinity. The *in vitro* evaluation of CaIX-P1-4-10 demonstrates improved binding and metabolic characteristics. However, organ distribution experiments still reveal low tumor-to-blood ratios and increased background noise, which is not favorable for imaging applications. Therefore, although our studies provide molecules with optimized *in vitro* properties, further experiments are necessary for generation of peptide-based ligands specifically targeting human carbonic anhydrase IX.

## Materials and Methods

### Chemical reagents and solvents

All reagents and solvents were purchased from commercial suppliers and were used without further purification.

### Cell lines

All cell lines were cultivated at 37°C in a 5% CO_2_ incubator. The human renal cell carcinoma cell line SKRC 52 was obtained by O. Boerman (Univ. of Nijmegen, The Netherlands). SKRC 52 was cultured in RPMI-1640 with GlutaMAX (Invitrogen) containing 10% (v/v) fetal calf serum (Invitrogen). BxPC3 was cultured in RPMI-1640 with extra D-Glucose (4.5 g/L) (Invitrogen) containing 10% (v/v) fetal calf serum.

### Peptide Synthesis

Peptides were prepared using standard Fmoc-based solid-phase peptide synthesis in an Applied Biosystems 433A synthesizer. 0.05 mmol Tentagel R RAM (Rapp Polymere Tübingen, Germany ) with a loading of 0.19 mmol/g and 0.5 mmol (10 eq.) of Fmoc-protected amino acids with the following side-chain protecting groups were used: tert-butyl (tBu) for Asp, Glu, Ser, Thr and Tyr, tert-butyloxycarbonyl (Boc) for Lys and Trp, trityl (Trt) for Asn, Gln and His, and 2,2,4,6,7-pentamethyldihydrobenzofuran-5-sulfonyl (Pbf) for Arg. The synthesis cycle of the Applied Biosystems 433A synthesizer consisted of: 1) Fmoc cleavage: 20% piperidine/DMF, 2) NMP washings, 3) coupling: Fmoc-AA-OH/HBTU/DIPEA/mmol peptidyl-resin 10/9/20/1, 8 min, 4) NMP washings.

Cleavage from the resin was performed with 95∶2.5∶2.5 TFA/water/triisopropylsilane for 1 h at room temperature and subsequent precipitation with cold diethyl ether. After a second precipitation with diethylether the residue was dried under reduced pressure. Purification of the synthesized peptides was performed by preparative RP-HPLC separation on a Gilson 321 pump HPLC system (Gilson Inc., Middleton, WI, USA) using a X-Bridge BEH C18 5 µm column (19×150 mm; Waters Corp., Millford Massachusetts, USA) with a gradient elution from 0.1% TFA in water to 0.1% TFA in acetonitrile, 0–35%B in 15 min (flow rate: 20 mL/min, absorbance: λ = 214 nm).

### HPLC analysis

Analytical reversed-phase high-performance liquid chromatography (RP-HPLC) was performed on an Agilent 1100 HPLC system using a Chromolith Performance RP-18e column (100×3 mm; Merck AG Darmstadt, Germany) eluted with a linear gradient (eluent A: 0.1% TFA in water; eluent B: 0.1% TFA in acetonitrile; flow rate: 2 mL/min, absorbance: λ = 214 nm).

### HPLC-MS analysis

All products of the peptide synthesis were further characterized by LC/MS using an Orbitrap Mass Spectrometer (Exactive, Thermo Fisher Scientific) coupled to an Agilent 1200 HPLC system on a Hypersil Gold C18 column (2.1×200 mm, 1.9m; Thermo Scientific, Bremen, Germany) eluted with a linear gradient (eluent A: 0.1% TFA in water; eluent B: 0.1% TFA in acetonitrile; 0–100% B in 30 min at 60°C, flow rate: 0.2 mL/min, absorbance: λ = 214 nm). Full scan single mass spectra (ESI, positive mode) were obtained by scanning from m/z = 200–4000.

### Radiolabeling of peptides

Radiolabeling with iodine-125 or iodine-131 was conducted at the tyrosine moiety of the peptide using the chloramine-T method a peptide solution in water (1 mM, 5 µL, 5 nmol) was added to phosphate buffer (0.25 M, pH 7.5, 25 µL). A solution of 1–15 MBq ^125^I-NaI or ^131^I-NaI in 10 µM NaOH was added and the labelling reaction was started by addition of an aqueous chloramine-T solution (10 mM, 10 µL). After 30 s, the labeling reaction was quenched by adding a saturated aqueous solution of methionine (10 µL).

### Radio-HPLC

Radio-HPLC was performed on an Agilent 1100 HPLC system equipped with a radioactivity detector (GABI Star, Raytest GmbH, Straubenhardt, Germany) using a Chromolith Performance RP-18e column (100×3 mm; Merck AG Darmstadt, Germany) eluted with a linear gradient (eluent A: 0.1% TFA in water; eluent B: 0.1% TFA in acetonitrile; 0–30% B in 10 min, flow rate: 2 mL/min).

Semi-preparative radio-HPLC separations were conducted on a Gyncotech P580 instrument equipped with a Shimadzu SPD 6-A multi-wavelength detector using a Chromolith performance RP-18e column (4.6×100 mm; Merck AG Darmstadt, Germany) eluted with a gradient (eluent A: 0.1% TFA in water; eluent B: 0.1% TFA in acetonitrile; 0–35% B in 10 min, flow rate: 2 mL/min, absorbance: λ = 214 nm).

### *In vitro* binding experiments

For binding experiments 5×10^5^ SKRC 52 or BxPC3 cells were seeded into 6-well plates and cultivated in 3 mL of incubation medium at 37°C for 24 h. After cell blocking with RPMI 1640 (without FCS) containing 1% BSA, the medium was replaced with 1 mL of fresh medium (without FCS) containing 0.8–1.2×10^6^ cpm of ^125^I-labeled peptide and incubation was performed for time periods varying from 10 min to 2 h at 37°C. To determine specific versus nonspecific binding, the cells were incubated with unlabeled competitors at concentrations varying from 10^−4^ to 10^−10^ mol/L. Octreotide was used as negative control competitor. After incubation the medium was removed and the cells were washed three times with 1 mL ice cold PBS in order to remove the unbound radiolabeled peptide. Subsequently, the cells were lysed with 0.5 mL NaOH 0.3 mol/L and the radioactivity was measured with a γ-counter. Bound radioactivity was calculated as percentage applied dose per 10^6^ cells. CA IX negative BxPC3 cells were used as negative control.

### Internalization studies

Subconfluent cell cultures of SKRC 52 cells were incubated with ^125^I-labeled peptide for 10, 30, 60, 120 and 240 min at 37°C and 4°C. Cellular uptake was stopped by removing the medium and washing three times with 1 mL PBS. Subsequently, cells were incubated with 1 mL of glycine-HCl, 50 mmol/L in PBS (pH 2.2) for 10 min at room temperature in order to remove the surface bound activity. The cells were then washed with 3 mL of ice-cold PBS and lysed with 0.5 mL of NaOH 0.3 mol/L. The surface and the internalized radioactivity were measured with a γ-counter and calculated as % applied dose/10^6^ cells.

### *In vitro* stability

^125^I-labeled peptide was incubated at 37°C on SKRC 52 and BxPC3 cells. At time points varying from 10 min to 2 h aliquots were taken and centrifuged for 5 min at 13,000 rpm. The supernatant was analyzed by HPLC with H_2_O∶CH_3_CN, 0–30% in 10 min.

### Serum stability assay

Metabolic stability was investigated in human serum (Sigma Aldrich, H4522). 5 MBq of radiolabeled peptide was dissolved in human serum (500 µL) and incubated at 37°C. The stability of the labeled peptide was monitored by analyzing aliquots (25 µl) via radio-HPLC at selected time points.

### Real time quantitative PCR

Total cellular RNA was isolated from confluent SKRC 52 and BxPC3 cells in 75 cm^2^ cell culture flasks using the Trizol method (TRIzol Reagent, Invitrogen). RNA extraction was carried out with a standard phenol-chloroform extraction and the concentration was measured with a NanoDrop spectrophotometer (ND-1000 PeqLab Biotechnologie GmbH, Germany). 500 ng was transcribed into DNA using M-MLV reverse transcriptase, 50 pmol random hexamer and 100 pmol of oligo(dT) primers (Promega, Madison, WI, USA). Quantification of relative mRNA transcript levels for CA IX was assessed on a StepOnePlus™ Real-Time PCR System (Applied Biosystems), applying the TaqMan methodology. Normalization was performed using GAPDH as housekeeping gene. Primers were obtained from Applied Biosystems (Foster City, CA, USA).

### Western Blot analysis

SKRC 52 and BxPC3 cells were grown to 80% confluency. Cells were washed twice with 10 ml ice-cold PBS pH 7,4 and scraped with a cell scraper. The lysate was transferred and centrifuged for 3 min at 1000 rpm. The pellet was washed once with 5 mL PBS and centrifuged for 3 min at 1000 rpm. The pellet was taken up in 2 mL of 1% Triton X-100. This mixture was centrifuged for 10 min at 2700 rpm.

Supernatant was collected for Western Blotting assay. Proteins were transferred from a polyacrylamide gel to a nitrocellulose membrane with a Mini Trans-Blotter (100 V for 90 min) in transfer buffer. After transfer, non-specific binding sites were blocked for 1 h with 5% non-fat milk powder in TBST buffer at room temperature. Rabbit IgG monoclonal anti-human CA IX antibody (abcam, ab108351; 1∶1000 dilution) was used as primary antibody. This was diluted in blocking buffer and incubated with the membrane overnight at 4°C. After washing in TBST, the nitrocellulose membrane was incubated with horseradish peroxidase conjugated antibody (R&D Systems, HAF008; 1∶1000 dilution) in blocking buffer at room temperature for 60 min. For the loading control, Anti-COX IV antibody (abcam, ab14744; 1∶5000 dilution) was used as primary antibody and for the secondary antibody Polyclonal Goat Anti-Mouse (DakoCytomation, P 0447; 1∶1000 dilution) was used. Antibody binding was determined using the enhanced chemiluminescence detection system according to the manufacturer's instructions. Exposures were recorded on hyperfilms for 10 sec to 3 min.

### *In vivo* organ distribution

Organ distribution studies were performed in 9-week-old female Balb/c nu/nu mice, carrying subcutaneously transplanted SKRC 52 tumors. Animals were obtained from Charles River WIGA and housed in VentiRacks (BioZone Global). For tumor transplantation, SKRC 52 cells were grown to 90% confluence, harvested with PBS/ethylene diamine tetraacetic acid, resuspended in Falcon Matrigel-Matrix (BD) and kept on ice. 200 µl of Matrigel-Matrix cell suspension were injected subcutaneously into the anterior region of the mouse trunk and the tumors were grown to a size of 1.0 cm^3^. ^131^I-labeled CaIX-P1-4-10 was injected into the tail vein of the animals (approximately 1 MBq) and at 15 min, 60 min and 240 min after injection the animals were sacrificed. Tumor, blood and selected tissues (heart, spleen, liver, kidney, muscle, intestinum and brain) were removed, drained of blood, weighed and the radioactivity was measured in a γ-counter (LB 951G; Berthold Technologies). Also 3 aliquots of the tracer solution used for injection were measured. The organ uptake was calculated as percentage injected dose per gram tissue (% ID/g).

All animal experiments were carried out in conformity with German law for protection of animals and are in compliance with European laws. Study approval was received by the Regierungspräsidium Karlsruhe, Abteilung 3, Baden-Württemberg, Germany, File reference: 35-9185.81/G-132/04.

### *In vivo* planar scintigraphy

Planar scintigraphy studies were performed in two Balb/c nu/nu mice, carrying subcutaneously transplanted SKRC 52 tumors. A cell suspension of 6×10^6^ cells in OPTI-MEM (Gibco, Invitrogen Life Technologies) was injected subcutaneously into the hind leg of the animals and the tumors were grown to a size of 1.0 cm^3^.^125^I-labeled CaIX-P1-4-10 peptide was injected into the tail vein of the animals (approximately 5 MBq) and at 10 min and 30 min p.i. planar scintigraphic images were acquired using a Gamma Imager (Biospace Lab)

### Statistics

Data were analyzed employing the paired two-tailed Student t-test and significance was assumed at P<0.05.

## Supporting Information

Figure S1 **Western Blot and RT-PCR analysis of SKRC 52 and BxPC3 cells.** Carbonic Anhydrase IX is expressed in the form of 54 and 58 kDa protein bands. COX IV was a loading control.
(TIF)
