# Supplementary figures and images for: Optimization of a Novel Peptide Ligand Targeting Human Carbonic Anhydrase IX
Source: PLoS One. 2012 May 31;7(5):e38279. doi: 10.1371/journal.pone.0038279 (PMC3365038; doi:10.1371/journal.pone.0038279)

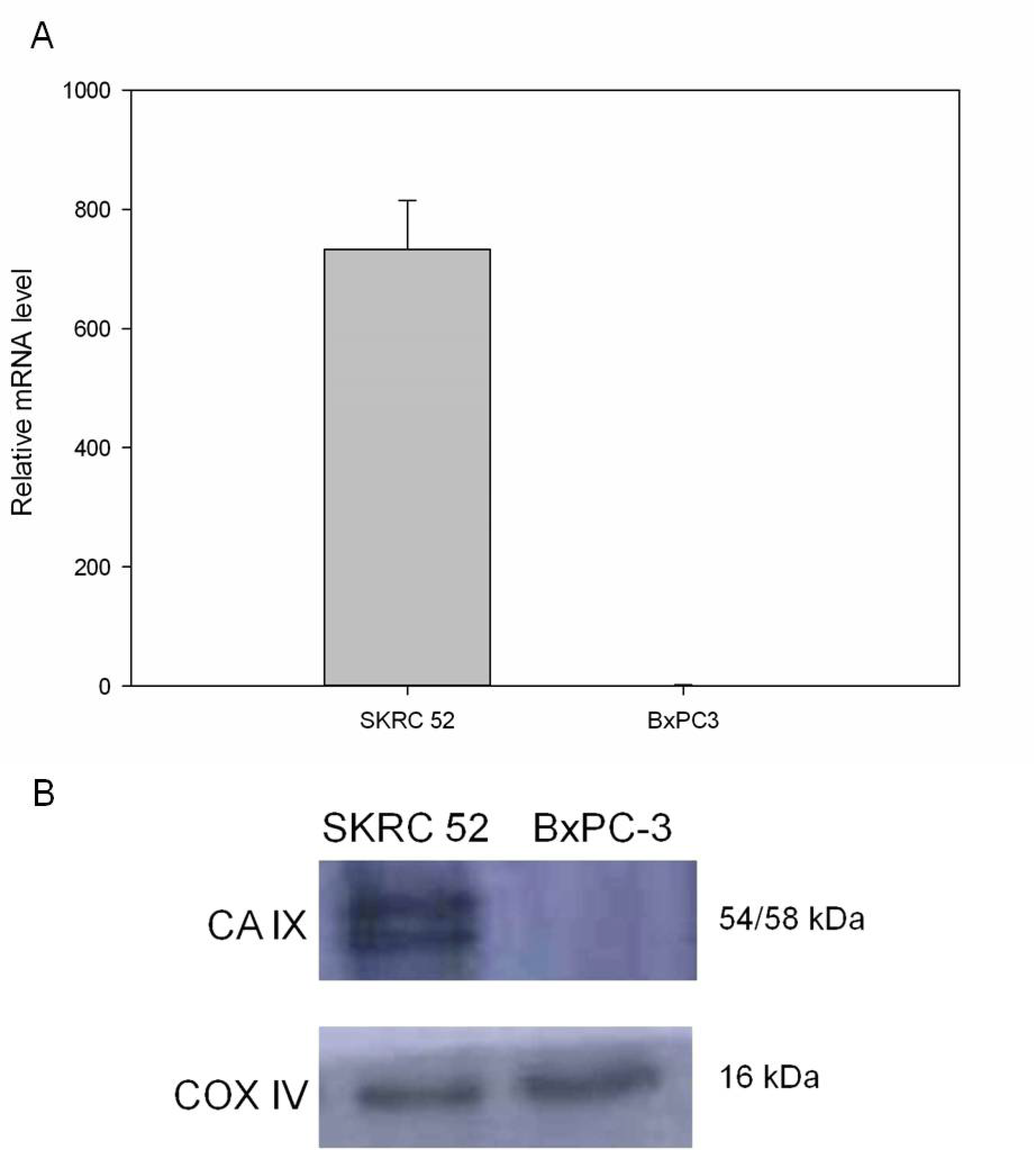

Supplement: Figure S1 — Western Blot and RT-PCR analysis of SKRC 52 and BxPC3 cells. Carbonic Anhydrase IX is expressed in the form of 54 and 58 kDa protein bands. COX IV was a loading control. (TIF) [file pone.0038279.s001.tif]
